# Green synthesis of chlorella-derived carbon dots and their fluorescence imaging in zebrafish†

**DOI:** 10.1039/d3ra07623g

**Published:** 2024-01-05

**Authors:** Yue Wang, Zhihi Gu, Jingyi Dong, Jie Zhu, Cunguang Liu, Guohan Li, Meichen Lu, Jian Han, Shengnan Cao, Liyong Chen, Wei Wang

**Affiliations:** a Key Laboratory of Applied Biology and Aquaculture of Northern Fishes in Liaoning Province, Dalian Ocean University Dalian 116023 China guzhizhi@dlou.edu.cn wangwei@dlou.edu.cn; b Department of Pharmaceutical Engineering, Bengbu Medical College Bengbu 233030 China

## Abstract

Recently, carbon dots (CDs) have been shown to exhibit exceptional water solubility, low toxicity, favorable biocompatibility, stable fluorescence properties with a wide and continuous excitation spectrum, and an adjustable emission spectrum. Their remarkable characteristics make them highly promising for applications in the field of bioimaging. Zebrafish is currently extensively studied because of its high genetic homology with humans and the applicability of disease research findings from zebrafish to humans. Therefore, spirulina, a commonly used feed additive in aquaculture, was chosen as the raw material for synthesizing fluorescent CDs using a hydrothermal method. On the one hand, CDs can modulate dopamine receptors in the brain of zebrafish, leading to an increase in dopamine production and subsequently promoting their locomotor activity. On the other hand, CDs have been shown to enhance the intestinal anti-inflammatory capacity of zebrafish. This study aimed to explore the chronic toxicity and genotoxicity of CDs in zebrafish while providing valuable insights for their future application in biological and medical fields.

## Abstract 2

Schematic illustration of fluorescence imaging and toxicology study of chlorella-derived CDs in zebrafish.

## Graphical abstract

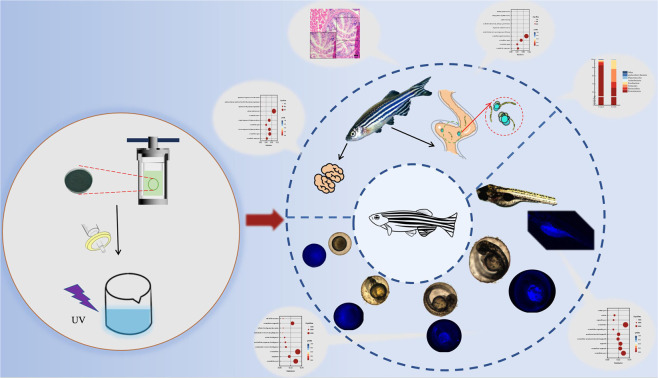

## Introduction

Carbon dots (CDs) have the advantages of adjustable photoluminescence, a large two-photon absorption cross section, easy functionalization, low toxicity, good chemical inertness, good dispersibility, and biocompatibility.^1,2^ When used in biomedicine, CDs not only can be used as a luminous indicator but also have high antibacterial efficiency.^3^ The precursors of CDs come from various sources, such as citric acid, small molecules, and plants.^4–8^ The marine environment provides numerous resources, including plants, animals, and microorganisms, which can be used to extract polysaccharides, such as alginate, carrageenan, chitin, chitosan, agarose, ulva, and porphyrin.^9^ These polysaccharides found in marine environments can serve as carbon-rich precursors for the synthesis of CDs.^10^ Using marine polysaccharides to produce CDs can transform renewable energy into cutting-edge technology products. At present, there is a concern that nanomaterials may cause intergenerational effects; therefore, the toxicity of nanomaterials to organisms has become a critical consideration before the application of nanomaterials.^11^

Chlorella, as a common health care product,^12,13^ also has anti-oxidation and anti-aging properties. It is often used as a dietary supplement in aquaculture^14^ since the addition of chlorella to fish diets improves enteritis caused by fish meal diets and improves fish performance against pathogens.^15^ At present, there are occasional reports about using chlorella to make bio-based CDs, which are often used for detection metal ions. CDs made by chlorella through a hydrothermal method were used to detect Fe^3+^ in wastewater,^16^ and it was found that they could be successfully doped without additional reagents, such as nitrogen, sulfur, phosphorus, or potassium. In addition, chlorella-derived CDs can also be used to distinguish the life and death of chlorella.^17^ Using chlorella-derived CDs to modify ZnO can be used for photocatalytic behavior in the degradation of carbamazepine.^18^

Zebrafish is a freshwater fish that has a small size, good vitality and reproductive ability, is easy to raise and manage,^19^ and has a short life cycle, going from fertilized eggs to adults in about three months,^20^ meaning they can be quickly raised in large-scale experiments. In addition, zebrafish larvae are transparent, so its internal structures and organ development process can be observed directly through the microscope. The use of zebrafish for toxicity assessment of CDs has the following advantages: zebrafish genome is 87% similar to the human genome; pathological states of many diseases and genes associated with disease causes are highly conserved among humans;^21,22^ compared with other model organisms zebrafish larvae are transparent, their internal structure and organ development process can be observed directly through the microscope, thus the transparency of zebrafish embryos and larvae provides experimental advantages for studying the accumulation sites of fluorescent-labeled CDs; Zebrafish's blood–brain barrier is similar to that of human, which can be used as the basis for drug screening in the central nervous system;^23^ Zebrafish's nervous system, including central nervous system and peripheral nervous system, is similar to that of human. Zebrafish has become a widely used model in behavioral neuroscience, especially as a disease model for Parkinson's disease (PD), Alzheimer's disease (AD), and depression;^24–26^ zebrafish has metabolic organs, such as liver, kidney, and intestine, and physiological structures similar to human beings.^27–29^

**Fig. 1:**
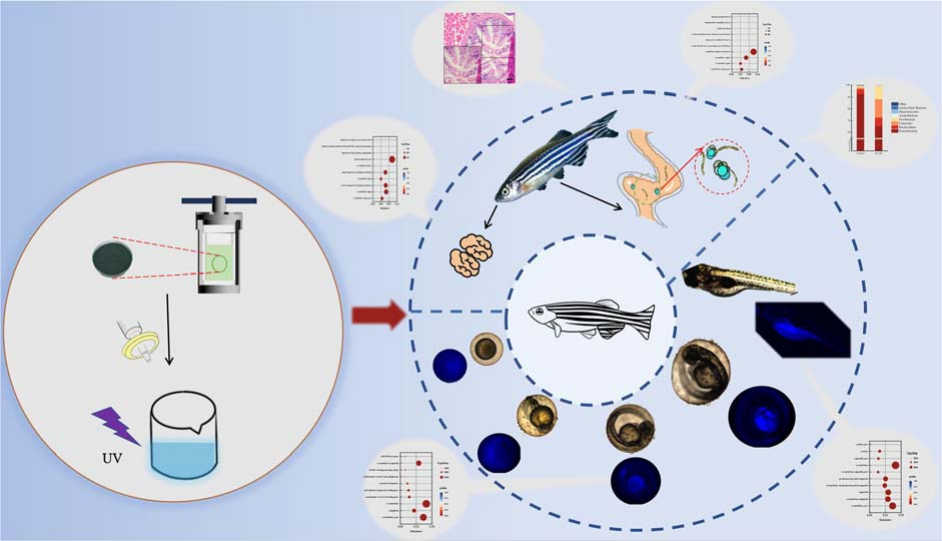
Schematic illustration of the fluorescence imaging and toxicology study of chlorella-derived CDs in zebrafish.

Chlorella was prepared into nano CDs, so that, on the one hand, the sterilization and anti-inflammatory effect of the CDs could be applied, while on the other hand, the size of the chlorella could be reduced to achieve the purpose of promoting absorption. However, there is no report on the application of chlorella-derived CDs to zebrafish. In order to study the possible effects of chlorella-derived CDs on organisms, herein, the eggs and adult fish of zebrafish were soaked with chlorella-derived CDs, and the effects of chlorella-derived CDs on zebrafish were studied by transcriptome sequencing and intestinal microbial sequencing (Fig. 1).

## Results and discussion

### Structural characterization of the CDs

**Fig. 2:**
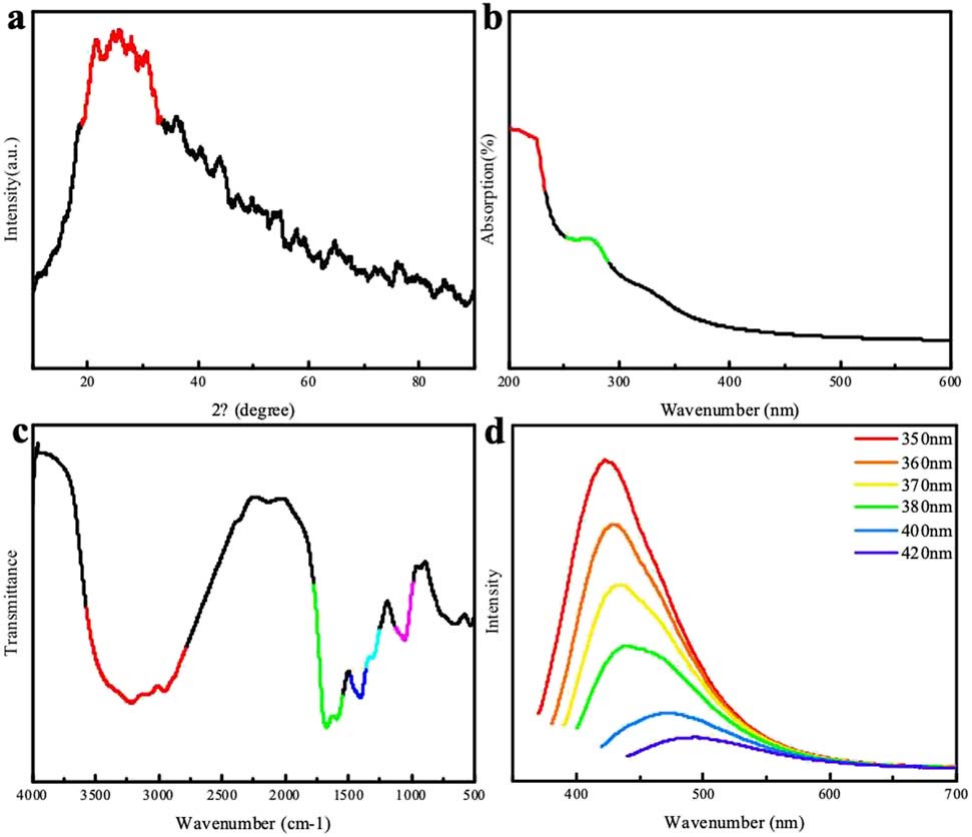
(a) XRD patterns of the CDs; (b) UV-visible absorption spectrum of the CDs; (c) FTIR spectrum of the CDs; (d) fluorescence spectra of the CDs.

The XRD patterns of the chlorella-derived CDs indicated the presence of graphitic carbon with a peak at approximately 24° (Fig. 2a). In the UV-vis absorption spectrum, the CDs showed a distinct peak around 280 nm (Fig. 2b). The excitation-dependent fluorescence of chlorella carbon was observed within an excitation wavelength range from 420 nm to 500 nm; with an increase in the excitation wavelength, there was a red-shift in the emission peak position. The CDs exhibited the strongest emission peak at 420 nm corresponding to blue fluorescence. Fourier-transform infrared spectroscopy analysis revealed that carbonyl groups (C=O) and hydroxyl groups (–OH) were present in the chlorella-derived CDs, as indicated by stretching vibration characteristic peaks at 1660 cm^−1^ and 3400 cm^−1^ (Fig. 2d), suggesting the good water solubility of the chlorella-derived CDs. Moreover, weak stretching vibration peaks of the C–O bond at 1100 cm^−1^, –CH_2_ at 2800 cm^−1^, and –CH_3_ 2900 cm^−1^ can be found, indicating the presence of a small amount of chlorella (Fig. 2c). The CD particles were very small, all smaller than 7 nm (Fig. S1†).

**Fig. 3:**
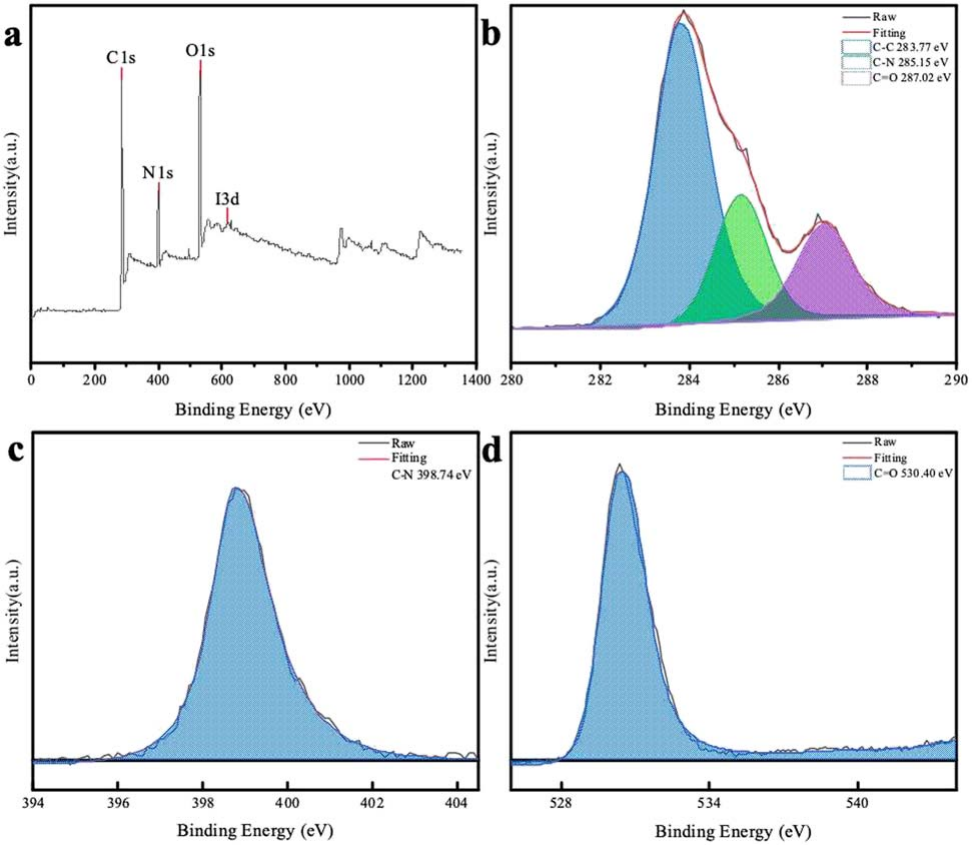
(a) XPS survey spectrum of the CDs and deconvoluted C 1s (b), N 1s (c), and O 1s (d) spectra of the CDs.

To further understand the chemical composition of the CDs, XPS spectrum of the chlorella-derived CDs were collected, and the three main binding energies of C 1s, N 1s, and O 1s peaks were at 283.77 eV, 398.74 eV, and 530.40 eV, respectively (Fig. 3a). This indicated that the CDs predominantly consisted of N, O, and C elements with a well-defined structure. The deconvoluted C 1s and O 1s core-level XPS spectra revealed the presence of C–C bonds and functional groups, such as C–N and C=O, in the CD domains (Fig. 3b and d). The N 1s XPS spectrum could be deconvoluted into one peak attributed to C–N (Fig. 3c).

### Fluorescence imaging and histological observations of the CDs in zebrafish

**Fig. 4:**
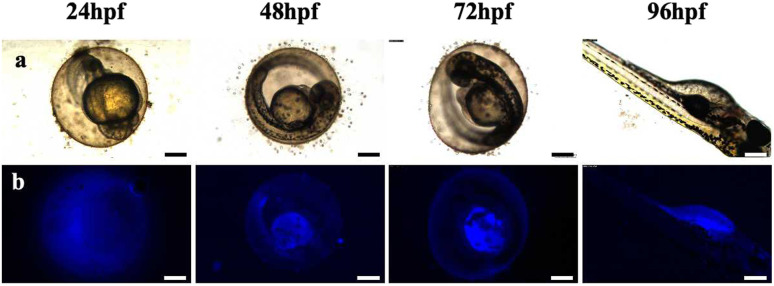
Metabolic processes of CDs in zebrafish from 24 to 96 hpf in CD solution (0.5 mg mL^−1^). (a) Bright field; (b) fluorescent field (ultraviolet) images. Scale bars, 250 μm.

Zebrafish exposed to CD solutions at concentrations of 0.5, 1, and 2 mg mL^−1^ exhibited excellent fluorescence imaging results (Fig. S2†). However, when exposed to high-concentration CD solution, a low survival rate was observed, which may be due to the high concentration of the CD solution and excessive color, which affected the circadian rhythm of the zebrafish eggs, thus affecting their growth and development. Notably, the mortality rate of zebrafish in the low-concentration CD solution (0.5 mg mL^−1^) showed no significant difference compared with the control group. Therefore, this concentration was selected for further exposure of the zebrafish during the fluorescence imaging and transcriptome experiments. The fluorescence intensity of the zebrafish eggs did not increase with prolonged exposure until reaching 96 hpf; instead, CDs accumulated within the yolk sac after hatching (Fig. S2†). To investigate the metabolic time of CDs in the zebrafish eggs, a blank solution was used as a replacement for the CD solution after soaking for 48 hpf for observation purposes. After discontinuing exposure for 48 hpf, there was a significant decrease in fluorescence intensity within the fry, indicating that the water-soluble CDs could not accumulate within the organisms (Fig. 4).

**Fig. 5:**
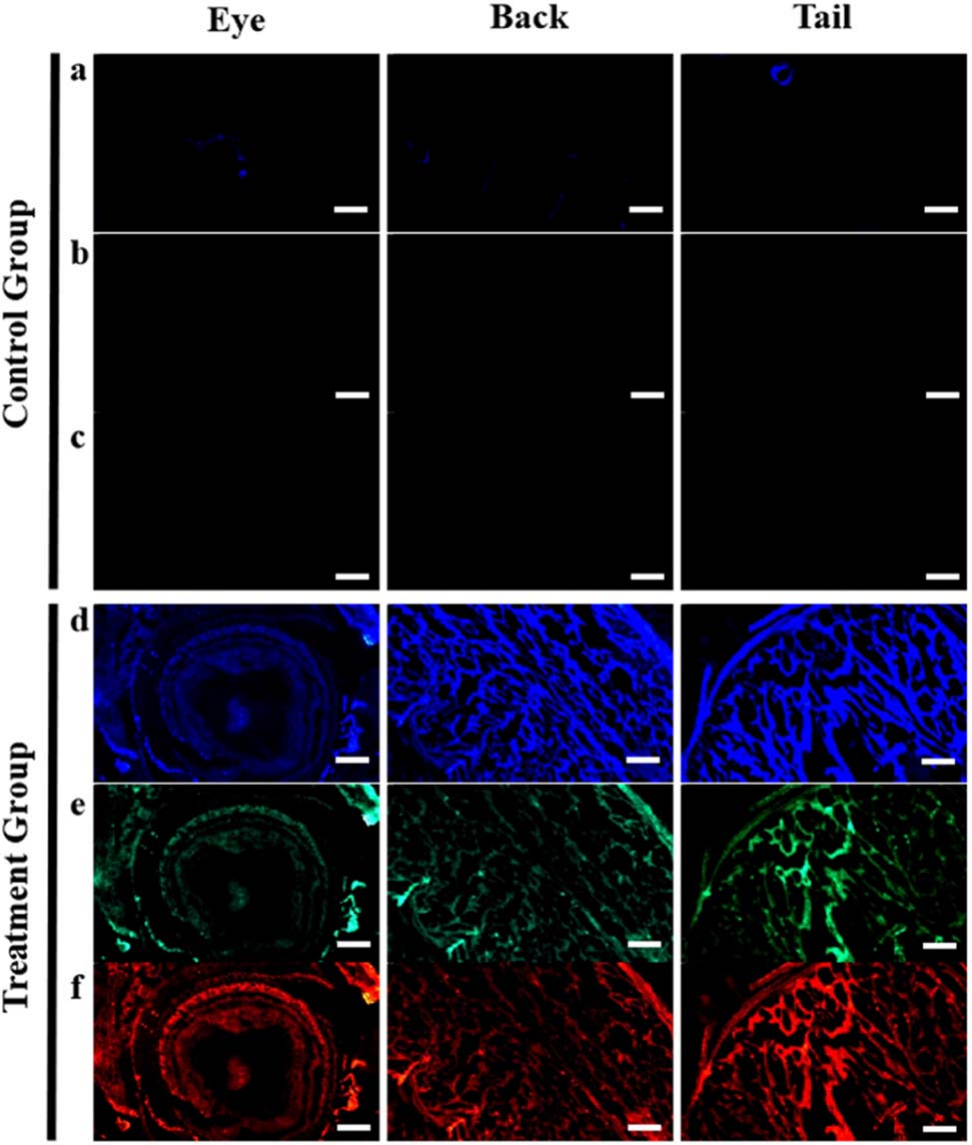
Fluorescence imaging of the eyes, back, and tail of zebrafish in CD solution (1 mg mL^−1^): (a) and (d) ultraviolet, (b) and (e) blue, and (c) and (f) orange. Control group (0 mg mL^−1^) of zebrafish for fluorescent imaging. Scale bars, 250 μm.

**Fig. 6:**
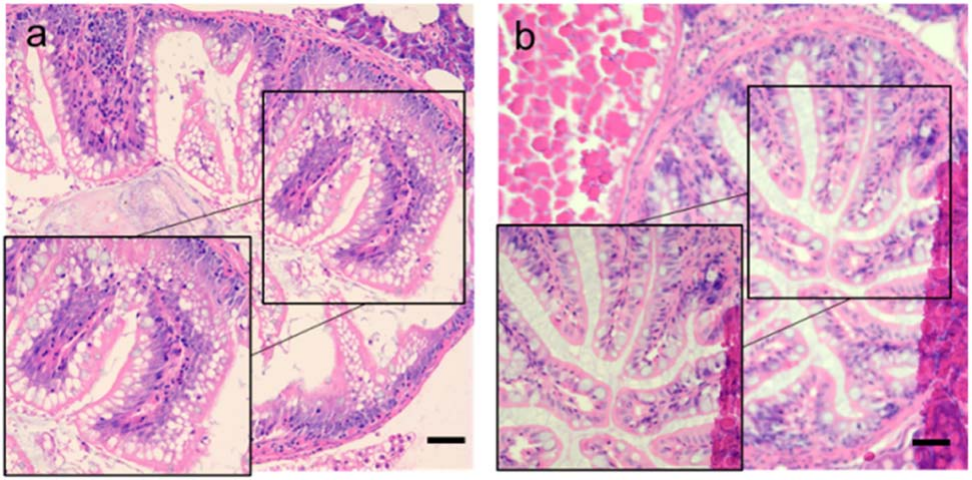
Intestinal sections for each group. (a) Control group (0 mg mL^−1^); (b) CDs solution (1 mg mL^−1^). Scale bars, 125 μm.

After soaking the zebrafish in CD solution (1 mg mL^−1^) for 12 h, it was observed that the CDs could diffuse into the fish through the eyes, back, and tail of the selected fish (Fig. 5). The control zebrafish developed severe enteritis, intestinal villi rupture, and cellular vacuolation; however, the cellular vacuolation and intestinal villi rupture decreased after immersion in CD solution (1 mg mL^−1^) for 12 h (Fig. 6). It is speculated that inflammatory lesions, such as intestinal wall rupture, gradually decreased in zebrafish under the influence of the CDs.

### Transcriptomic analysis of zebrafish

High-throughput sequencing was employed for the transcriptome sequencing analysis of the zebrafish eggs exposed for 48 hpf (experimental group named CDs-S) and 96 hpf (experimental group named CDs-L). The sequencing results revealed the upregulation of more genes in the zebrafish eggs under short-term exposure to CD solution. With the prolonged exposure time, differential gene expression increased and became predominantly upregulated (Fig. S3†). The differentially expressed genes induced by CD exposure in zebrafish eggs were primarily associated with metabolism-related pathways. Lipid metabolism and amino acid metabolism may be affected by exposure to the CD solution in these eggs. In addition, some genes were concentrated within genetic information processing-related pathways, suggesting a potential impact on genetic information processing in zebrafish eggs.

Both GO and KEGG databases were used to select the 10 pathway items with the most significant enrichment to be displayed in the figure. If there were fewer than 10 enriched pathway items, all of them were displayed (Fig. S4 and S5†). The CDs-S differential genes were predominantly enriched in 651 GO functional categories, with a particular concentration in the extracellular region, extracellular space, and formation of the primary germ layer. The CDs-L differential genes exhibited enrichment across 817 GO functional categories, primarily within organelles and intracellular compartments. The CDs-S group was mainly enriched in endocytosis (Fig. S4c†). CDs-L differential genes were significantly enriched in four pathways: spliceosome, protein processing in the endoplasmic reticulum, RNA transport, and ribosome biogenesis in eukaryotes. It is speculated that short-term exposure to CD solution may impact protoderm formation in eggs, while prolonged exposure could induce changes in egg organelles (Fig. S4d†).

During the experiment, it was found that CDs may affect the Wnt signaling pathway of fish eggs. Most of the genes in the signaling pathway were down-regulated when soaked for a short time, while most of the genes were up-regulated when soaked with CDs for a long time. Wnt is associated with zebrafish embryonic development,^30^ and Wnt/β-catenin signaling is essential for early fish swim bladder development,^31^ while Wnt may also affect zebrafish brain development.^32^

Adult zebrafish exposed to CDs (1 mg mL^−1^) for 12 h were selected for transcriptome sequencing and intestinal flora sequencing of the brain (referred to as CDs-B) and intestine (referred to as CDs-I). In response to CD exposure, zebrafish brain tissue exhibited fewer differentially expressed genes, which were mostly down-regulated and enriched in metabolic pathways related to dopamine secretion, indicating a potential decrease in dopamine levels (Fig. S5a and b†) when exposed to >200 μg mL^−1^ CD solution. The CDs-B group showed enrichment in 112 GO functional categories, specifically concentrated in the extracellular region and cell cycle. The CDs-I group displayed enrichment across 130 GO functional categories, particularly those associated with immune system processes and the extracellular region. The differential genes were enriched, and the KEGG enrichment point diagram of the differential genes graphically displays the KEGG enrichment analysis results.

The differential genes of the CDs-B group are enriched in 11 metabolic pathways, most of which are enriched in MAPK signaling pathway, dopaminergic synapse. The CDs-B differential genes were enriched in one metabolic pathway, and were enriched in the PSteroid hormone biosynthesis in the endoplasmic reticulum (Fig. S5c†). The CDs-I group differential genes were enriched in 7 metabolic pathways, and they were also enriched in cytokine–cytokine receptor interaction, protein digestion and absorption, glycerophospholipid metabolism, *etc* (Fig. S5d†).

Compared with the control group, the CDs affected the intestinal flora abundance of zebrafish. The highest abundance was proteobacteria (92.86%), and the highest abundance of CDs-I group was proteobacteria (65.33%) and firmicutes (15.89%) (Fig. S6†). As can be seen from the results of α diversity analysis, the Chao1, Shannon, and Simpson indexes all showed an increase in intestinal flora diversity in the CDs-I group. The influence of CDs on the intestinal flora of zebrafish was mainly reflected in the diversity of intestinal flora. After soaking in CDs, the diversity of the intestinal flora of zebrafish increased, while the abundance of proteobacteria in the intestinal flora decreased, while the abundance of fusobacteria increased.

## Conclusions

The fluorescence imaging results of zebrafish demonstrated that chlorella-derived CDs exhibited a pronounced fluorescence imaging effect. Within a specific concentration range, the CDs did not significantly impact the survival data of zebrafish adults and eggs. However, at the molecular level, the CDs influenced the brain, intestine, and eggs. It was observed that the carbon dots (CDs) exhibited excellent fluorescence imaging capabilities in zebrafish, indicating their favorable biocompatibility. Transcriptomics and 16S gene analysis were employed to investigate the molecular-level effects of the CDs on zebrafish embryo development, brain tissue function, and intestinal tissue health. The findings revealed a significant impact of CDs on the dopaminergic pathway in the zebrafish brain. By up-regulating the D2 receptor within this pathway, the CDs induced K^+^ channel opening, resulting in heightened fish activity levels and enhanced external performance. The transcriptome sequencing of zebrafish intestinal tissues demonstrated that the CDs promoted complement coagulation cascade activation within the intestine, suggesting potential anti-inflammatory effects. Furthermore, the analysis of the intestinal flora indicated that the CDs increased diversity and improved the overall environment of gut microbiota. This study aimed to explore chronic toxicity and genotoxicity associated with the CDs in zebrafish while providing valuable insights for their application in biology and medicine. Through the analysis of the zebrafish gut microbiota sequencing data, it was observed that the CDs could augment the diversity of intestinal flora and establish a new equilibrium. The affected pathway of the CDs may inspire the development of chlorella-derived CDs as gene-related inhibitors or promoters, thereby offering new perspectives for future nano-drug applications.

## Ethical statement

All animal procedures were performed in accordance with the Guidelines for Care and Use of Laboratory Animals of Dalian Ocean University and approved by the Animal Ethics Committee of Dalian Ocean University.

## Author contributions

Conceptualization and methodology: Zhihi Gu and Jie Zhu; investigation, visualization, data collection, and validation: Yue Wang, Jingyi Dong, Guohan Li, Cunguang Liu, and Jian Han; formal analysis: Meichen Lu, Shengnan Cao, and Liyong Chen; writing-original draft preparation: Zhihi Gu, Yue Wang, and Wei Wang; and funding acquisition, supervision, writing-review, and editing: Zhihi Gu.

## Conflicts of interest

There are no conflicts to declare.

## Supplementary Material

RA-014-D3RA07623G-s001
