# Correction: Green synthesis of chlorella-derived carbon dots and their fluorescence imaging in zebrafish

**DOI:** 10.1039/d4ra90048k

**Published:** 2024-05-02

**Authors:** Yue Wang, Zhizhi Gu, Jingyi Dong, Jie Zhu, Cunguang Liu, Guohan Li, Meichen Lu, Jian Han, Shengnan Cao, Liyong Chen, Wei Wang

**Affiliations:** a Key Laboratory of Applied Biology and Aquaculture of Northern Fishes in Liaoning Province, Dalian Ocean University Dalian 116023 China guzhizhi@dlou.edu.cn; b Department of Pharmaceutical Engineering, Bengbu Medical College Bengbu 233030 China

## Abstract

Correction for ‘Green synthesis of chlorella-derived carbon dots and their fluorescence imaging in zebrafish’ by Yue Wang *et al.*, *RSC Adv.*, 2024, **14**, 1459–1463, https://doi.org/10.1039/D3RA07623G

The authors regret that the name of one of the authors (Zhizhi Gu) was shown incorrectly in the original article. The corrected author list is as shown above.

The Royal Society of Chemistry apologises for these errors and any consequent inconvenience to authors and readers.

## Supplementary Material

