# Supplementary material for: Green synthesis of chlorella-derived carbon dots and their fluorescence imaging in zebrafish
Source: RSC Adv. 2024 Jan 5;14(2):1459–63. doi: 10.1039/d3ra07623g (PMC10768801; doi:10.1039/d3ra07623g)
Supplement: RA-014-D3RA07623G-s001 [file RA-014-D3RA07623G-s001.pdf]

Electronic Supplementary Material (ESI) for RSC Advances.
This journal is © The Royal Society of Chemistry 2023

Electronic Supplementary Information

Green synthesis of chlorella-derived carbon dots and their fluorescence imaging in zebrafish

Yue Wang,[a] Zhihi Gu,*[a] Jingyi Dong,[a] Guohan Li,[a] Meichen Lu,[a] Jie Zhu,[a], Cunguang Liu,[a] Jian Han,[a] Shengnan Cao,[a] Liyong Chen [b] and Wei Wang*[a]

[a] Key Laboratory of Applied Biology and Aquaculture of Northern Fishes in Liaoning Province, Dalian Ocean University, Dalian, Dalian 116023, China. E-mail: guzhizhi@dlou.edu.cn

[b] State Key Laboratory of Fine Chemicals Dalian University of Technology 2 Linggong RD., Dalian, 116024, China.

Experimental Section

Materials and Chemicals

All chemical reagents were used as received. Chlorella were purchased from Xinyuan Fine Chemical Co., LTD., China. Zebrafish were purchased from Shanghai Feixi Co., LTD..

Characterization Methods

X-ray diffraction (XRD) was conducted with a Rigaku D/Max 2400 automatic powder X-ray diffractometer with Cu-Kα radiation (λ = 1.5418 Å). UV-Vis absorption spectroscopy was performed on U-4100 spectrophotometer. Surface elemental composition and chemical state of samples were analyzed by X-ray photoelectron spectroscopy (XPS) on Thermo ESCALAB 250Xi with Al- K α radiation (hν = 1486.6 eV). Fourier transform infrared (FTIR) spectroscopy was carried out on a Nicolet 6700 spectrometer. Raman spectroscopy was conducted on a Jobin Yvon LabRAM HR Evolution Raman spectrometer.

Synthesis of CDs

Chlorella (10 g) and distilled water (50 mL) were added into the beaker sequentially, and transferred the solution to a reactor after stirring for 10 min. The reactor was heated at 200 °C for 8 h. Afterward, the final mixture was cooled down to room temperature and collected by suction filtrating using a pinhole filter (0.22 μm) before freeze drying of the resulting brown powder sample.

Breeding conditions of zebrafish

The male/female zebrafish were separated. Water was changed daily, and zebrafish were fed three times a day. The light period of 12 h was temporarily extended for 7 days. The male and female fish were placed in the middle of the spawning box at opposite ends of the board. At 8 am the next day, the cover was removed, and two hours later, the eggs were collected.

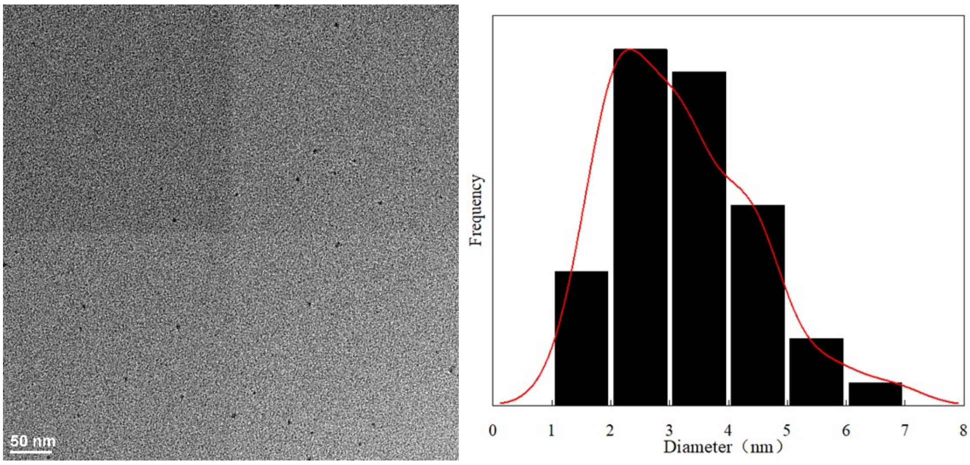

Figure S1. (a) TEM of CDs; (b) the corresponding particle size distribution map.

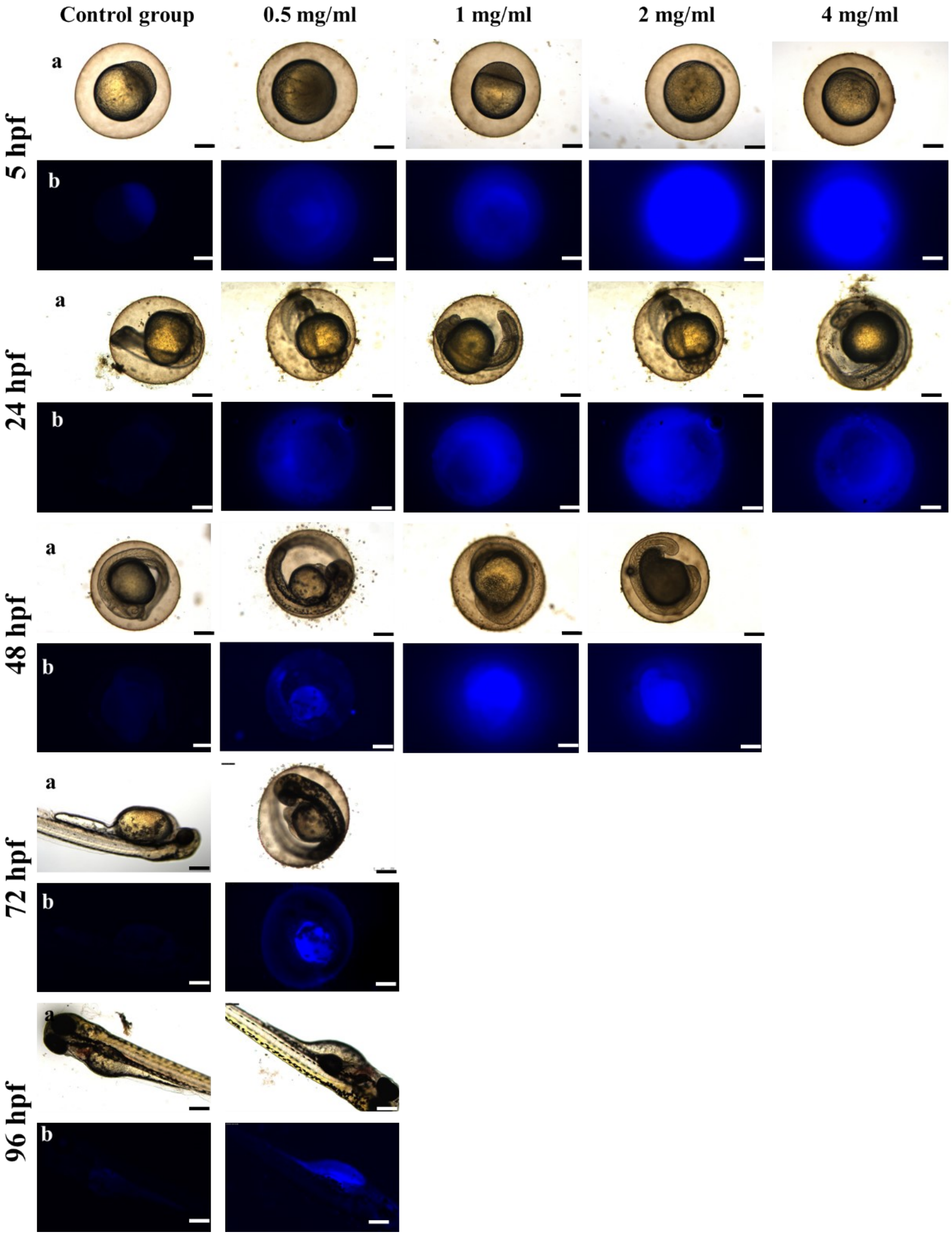

Figure S2. The fluorescent imaging of zebrafish eggs in different concentrations of CDs solution (0, 0.5, 1, 2, and 4 mg/mL). (a) bright field; (b) fluorescent field (ultraviolet). Scale bars, 250 μm.

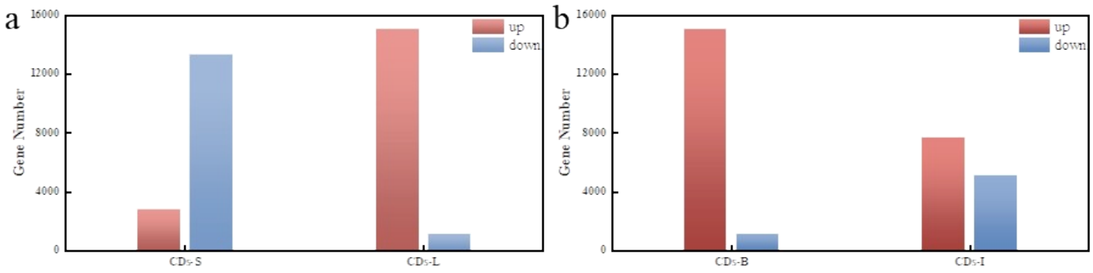

Figure S3. Differential gene up-regulation and down-regulation. Exposed time of 48 hpf (experimental group named CDs-S) and 96 hpf (experimental group named CDs-L).

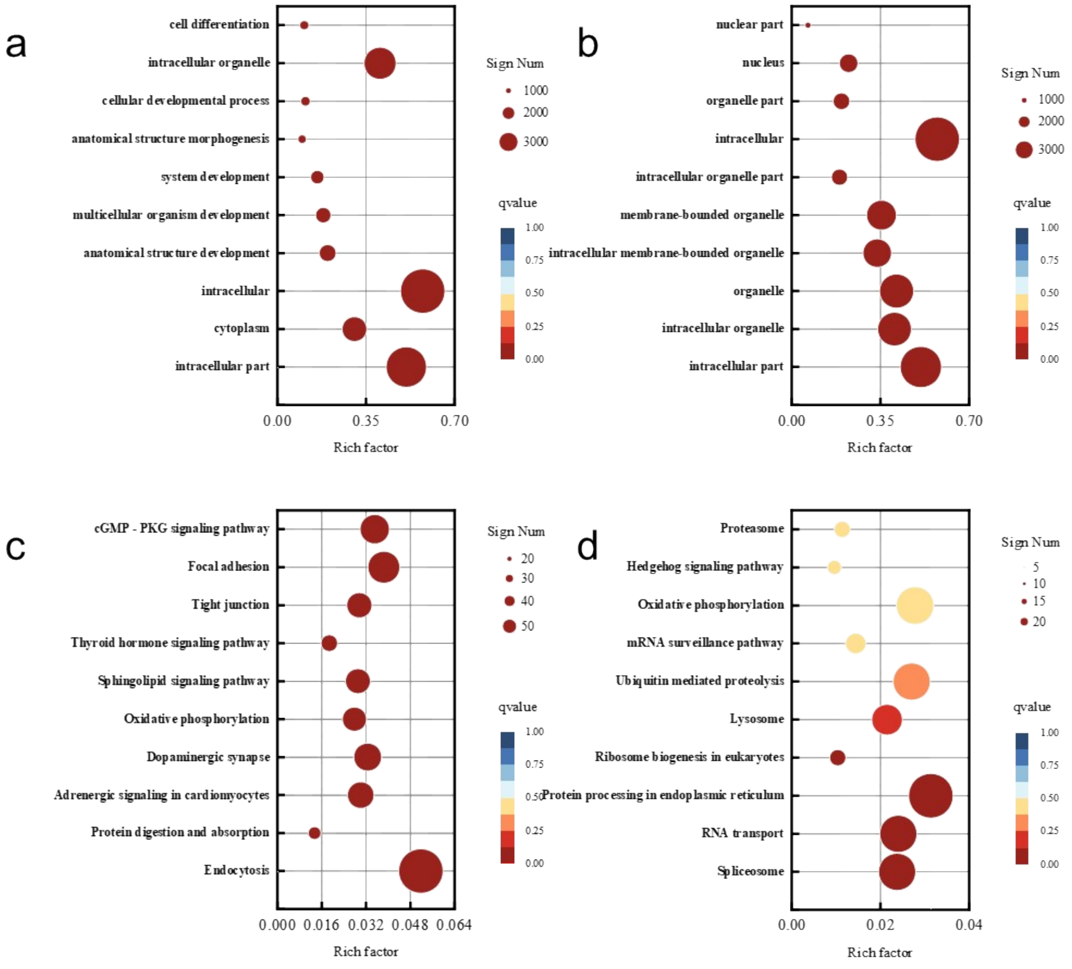

Figure S4. Zebrafish eggs exposed to CDs (0.5 mg/mL) were selected for transcriptome sequencing. GO enrichment plot of CDs-S (a) and CDs-L (b); KEGG enrichment plot of CDs-S (c) and CDs-L (d). Exposed time of 48 hpf (experimental group named CDs-S) and 96 hpf (experimental group named CDs-L).

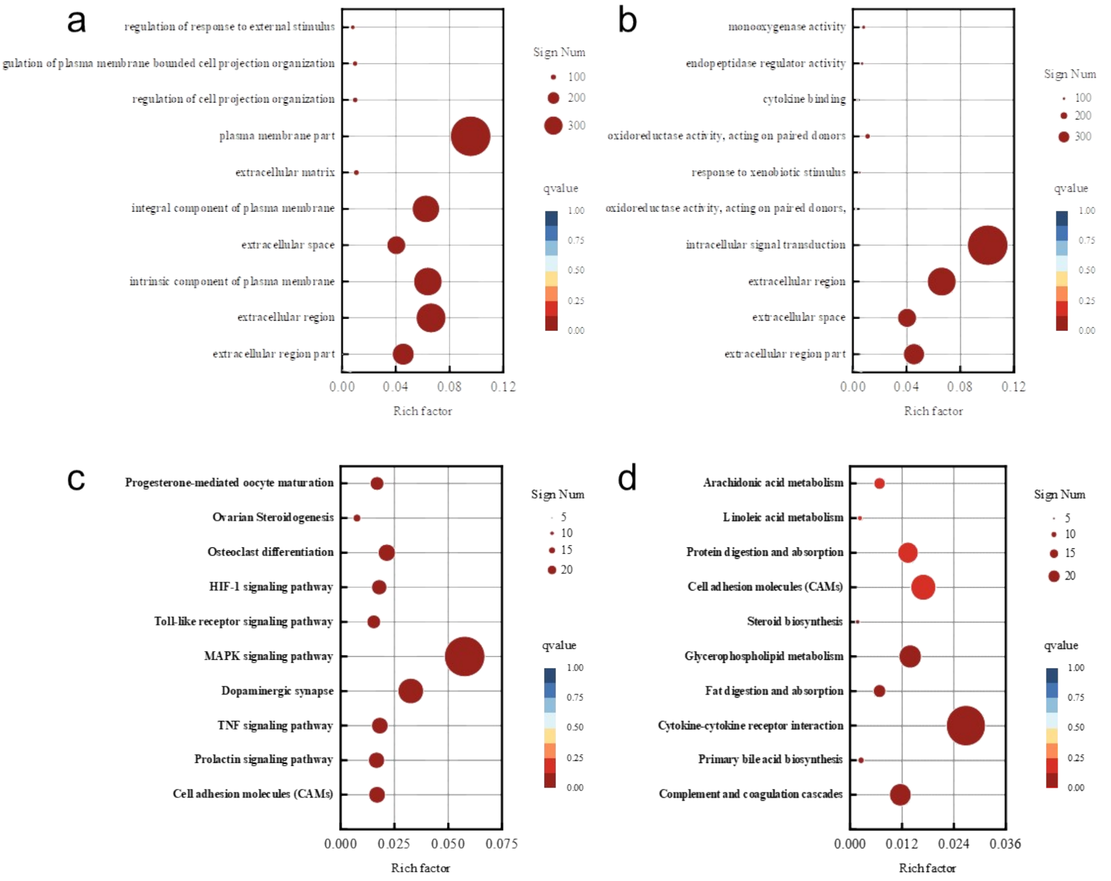

Figure S5. Adult zebrafish exposed to CDs (1 mg/mL) for 12 h were selected for transcriptome sequencing of the brain (referred to as CDs-B) and intestine (referred to as CDs-I). GO enrichment plot of CDs-B (a) and CDs-I (b); KEGG enrichment plot of CDs-B (c) and CDs-I (d).

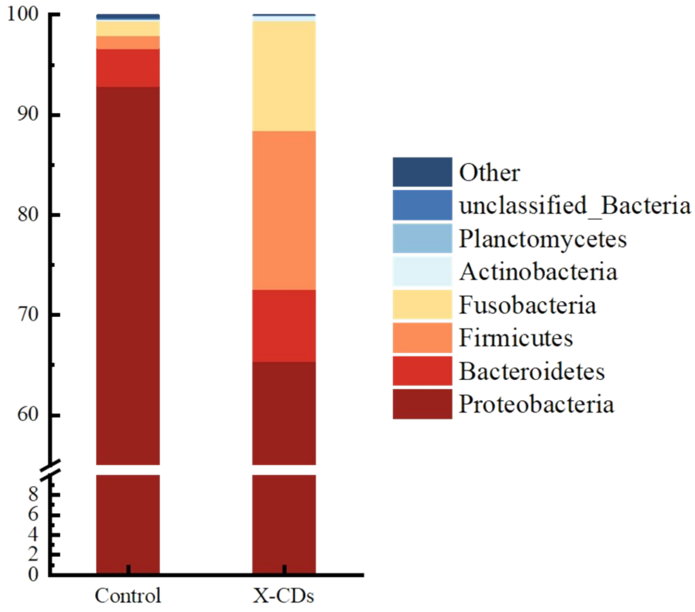

Figure S6. Intestinal flora abundance map of zebrafish after CDs solution (1 mg/mL) exposure for 12h.
